# Hematological Versus Clinical Predictors of Tumor Grade in Endometrial Cancer: A Comparative Machine Learning Study with SHAP-Based Explainability

**DOI:** 10.3390/diagnostics16182987

**Published:** 2026-09-15

**Authors:** Esra Akaydın Gültürk, Şerife Özlem Genç

**Affiliations:** 1 Department of Biostatistics, Faculty of Medicine, Sivas Cumhuriyet University, 58140 Sivas, Türkiye; 2 Department of Obstetrics and Gynecology, Faculty of Medicine, Sivas Cumhuriyet University, 58140 Sivas, Türkiye

**Keywords:** endometrial neoplasms, neoplasm grading, systemic inflammation, neutrophil-to-lymphocyte ratio, machine learning, explainable artificial intelligence, SHAP

## Abstract

**Background**: Systemic inflammatory indices derived from routine complete blood counts have been proposed as inexpensive biomarkers in gynecological malignancies. Their ability to discriminate tumor grade in endometrial cancer, however, has rarely been evaluated against routinely available clinical variables under rigorous validation. **Methods**: We retrospectively analyzed 225 women with endometrial cancer (166 low-grade, G1–G2; 59 high-grade, G3). Nine inflammatory indices were computed. Four feature sets were compared using a prespecified elastic-net logistic regression under repeated nested cross-validation (5 outer × 5 inner folds, 20 repetitions) with 5000 stratified bootstrap confidence intervals: a collinearity-reduced inflammatory panel, the full inflammatory panel, preoperative clinical variables (age, CA-125 and preoperative albumin), and their combination. Selection among nine classifiers was retained as an exploratory analysis. Because 66% of high-grade events were non-endometrioid, every analysis was repeated in three cohorts: the full cohort, endometrioid tumors only, and the subgroup with assessed hormone receptor status. Histologic subtype was deliberately excluded because serous, clear-cell and undifferentiated carcinomas are high-grade by definition. Model behavior was interpreted with TreeSHAP and cross-checked against permutation importance. **Results**: Of nine inflammatory indices, only the eosinophil-to-lymphocyte ratio remained statistically associated with grade after false discovery rate correction (AUC 0.626, 95% CI 0.545–0.707; q = 0.036), with all others showing negligible effect sizes (|r| < 0.08); its discrimination was nonetheless modest and insufficient for individual-level prediction. The inflammatory panel achieved AUC 0.533 (95% CI 0.443–0.621), within the range attainable by chance in this design (permutation *p* = 0.254). Preoperative clinical variables reached AUC 0.687 (95% CI 0.590–0.772) in the full cohort but 0.390 (0.257–0.524) in endometrioid tumors alone and 0.627 (0.485–0.765) in the receptor-assessed subgroup. Adding inflammatory indices to clinical variables did not improve discrimination (ΔAUC = −0.025, 95% CI −0.062 to +0.012; one-sided upper bound +0.007 against a prespecified margin of +0.05). The results were unchanged under an alternative grade dichotomization and after excluding patients with inconsistent blood-count entries. **Conclusions**: Most inflammatory indices did not demonstrate clinically useful discrimination between G3 and G1–G2 disease in this cohort, and did not add incremental discriminative value beyond the prespecified margin over routinely available preoperative clinical variables. The apparent discrimination of the clinical variables in the full cohort did not survive restriction to endometrioid histology, indicating that it reflected histologic composition rather than grade. Decision curve analysis on recalibrated probabilities showed that no model exceeded the treat-all strategy at thresholds below the prevalence of high-grade disease, and that, above it, any advantage over treating no one was small in the full cohort and absent within endometrioid tumors.

## 1. Introduction

Endometrial cancer is the most frequently diagnosed gynecological malignancy in developed countries and represents a substantial threat to women’s health [1]. Accurate staging and early diagnosis directly influence disease course and treatment success. Within this framework, tumor grade occupies a central position in clinical management, providing information about tumor aggressiveness and prognosis.

Low-grade tumors generally follow a less invasive course, whereas high-grade tumors may behave more aggressively and require more intensive treatment strategies [2,3]. Preoperative endometrial sampling remains the cornerstone of histological grading. Discordance between biopsy-based grade and final hysterectomy pathology is nonetheless consistently reported, with upgrading described in 3.6% of preoperative G1 and 11.4% of preoperative G2 tumors in one series of 335 endometrioid cancers [4]. Discordance in the opposite direction is also reported and remains clinically relevant: in a recent series of FIGO stage I endometrioid carcinomas graded high on preoperative biopsy, a substantial proportion proved low-grade on the hysterectomy specimen [5]. Readily available preoperative parameters capable of complementing biopsy findings may therefore contribute to preoperative risk stratification.

The role of the systemic inflammatory response in cancer development and progression has become increasingly recognized. Hematological markers such as the neutrophil-to-lymphocyte ratio (NLR), platelet-to-lymphocyte ratio (PLR), monocyte-to-lymphocyte ratio (MLR) and the pan-immune-inflammation value (PIV) have been investigated as prognostic indicators across several tumor types [6,7]. Because these markers are inexpensive and derivable from a routine complete blood count, they have attracted considerable interest for clinical use. These reports address four distinct questions that are not interchangeable. The first concerns prognosis: meta-analyses in endometrial and gynecological cancer report associations between the systemic immune-inflammation index and survival endpoints [8,9], and single-center series report similar associations for the neutrophil-to-lymphocyte and platelet-to-lymphocyte ratios [10,11]. The second concerns diagnosis, that is, the discrimination of endometrial malignancy from benign uterine disease, where the indices perform moderately [7]. The third concerns association with clinicopathological features such as depth of myometrial invasion [12]. The fourth—preoperative discrimination of G3 from G1–G2 disease—is the question of the present study; among the primary studies appraised in Supplementary Table S1, we found none that addresses it directly, grade appearing in those reports only as one covariate among several rather than as the prediction target. Across the primary studies appraised in Supplementary Table S1, correction for multiple comparisons across the indices tested, formal assessment of multicollinearity among algebraically related indices, and nested or external validation of predictive models were frequently absent. Whether these markers carry information about tumor grade that is not already contained in routinely available clinical variables therefore remains an open question.

Machine learning (ML) algorithms can integrate multiple clinical and laboratory parameters and have been applied to risk assessment in endometrial cancer [13,14]. Explainable artificial intelligence (XAI) approaches, in particular SHAP (SHapley Additive exPlanations), render model decisions transparent and have been used to increase clinical trust in such models [15]. Importantly, explainability methods describe how a model uses its inputs; they do not establish that the model discriminates better than chance. Reporting feature attributions without first demonstrating adequate discrimination is a recurrent shortcoming in the applied XAI literature.

The present study was designed as a direct comparison rather than as a model development exercise. We asked three questions: (i) Do systemic inflammatory indices discriminate high-grade from low-grade endometrial cancer? (ii) How does their performance compare with that of routinely available preoperative clinical variables? (iii) Do inflammatory indices add incremental discriminative value when combined with clinical variables? Analyses were conducted with explicit control of multiple comparisons, formal multicollinearity diagnostics, nested cross-validation, permutation-based null modeling, and SHAP-based explainability cross-validated by permutation importance.

The aim of the present study is not to replace preoperative endometrial sampling but to ask whether clinical and hematological data already available before surgery can help estimate the probability that the hysterectomy specimen will show G3 rather than G1–G2 disease. Preoperative biopsy grade is the current clinical standard and the relevant comparator throughout.

## 2. Materials and Methods

### 2.1. Study Design and Population

This single-center, retrospective, cross-sectional study was conducted at the Department of Obstetrics and Gynecology, Faculty of Medicine, Sivas Cumhuriyet University, Sivas, Türkiye. The study population comprised all consecutive women who underwent surgical treatment for endometrial cancer at this department between January 2011 and December 2021. All demographic, clinical, laboratory, and histopathological data were retrieved retrospectively from the hospital information management system and archived medical records.

**Figure 1:**
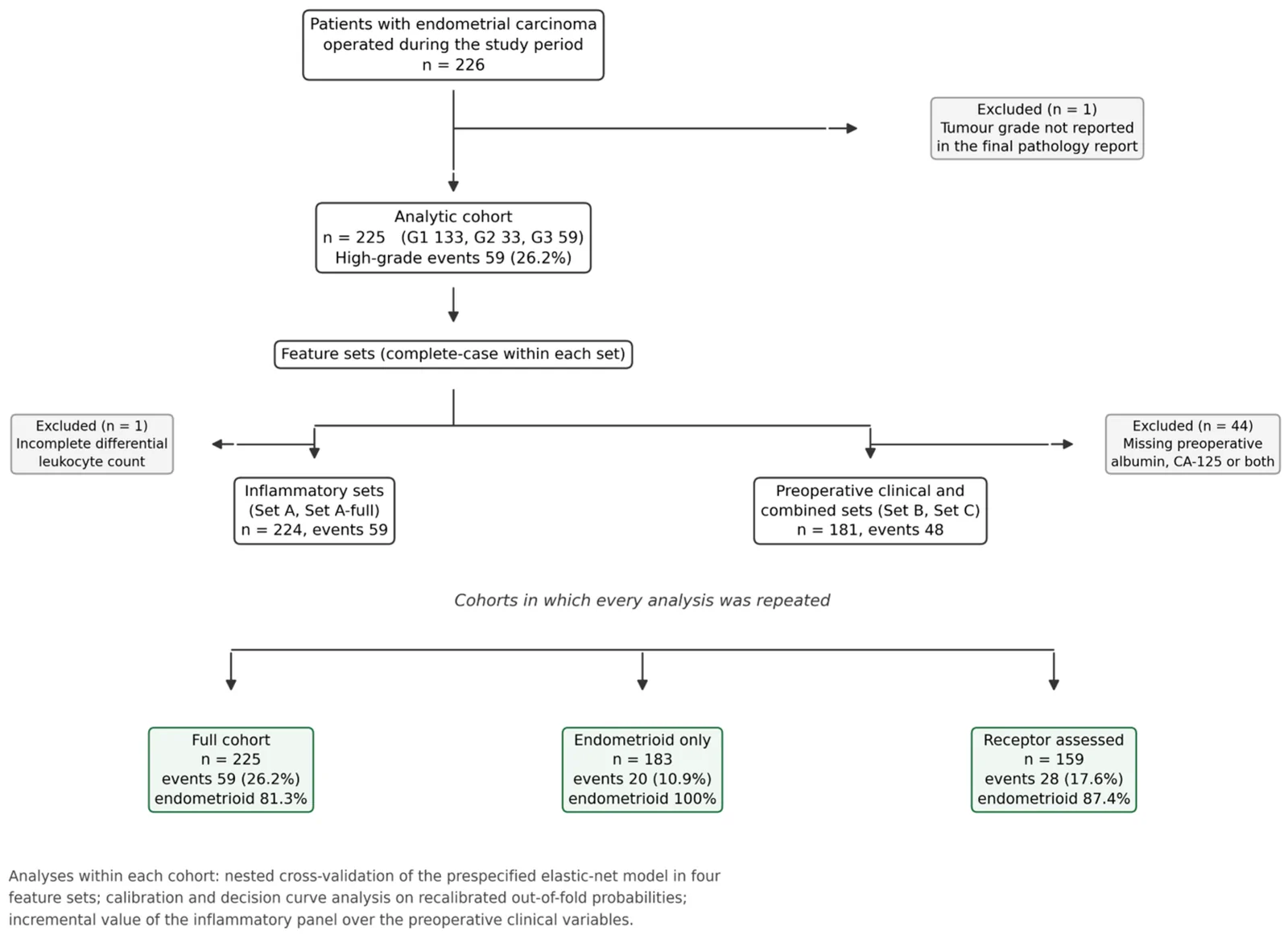
Participant flow. Patients identified, reasons for exclusion, the analytic cohort, the number of patients contributing to each feature set, and the three cohorts in which every analysis was repeated.

Women were eligible if they had a histopathologically confirmed diagnosis of endometrial carcinoma, underwent surgery at the study center during the study period, and had a preoperative complete blood count on record. Patients of all histologic subtypes were included. Of the 226 patients identified, one was excluded because tumor grade was not reported in the final pathology report, leaving 225 patients for analysis (Figure 1). Tumors were dichotomized according to the WHO 2020 classification of female genital tumors [16] into low-grade (G1–G2; n = 166, 73.8%) and high-grade (G3; n = 59, 26.2%). No exclusion was applied for concomitant infection, corticosteroid use or chronic inflammatory comorbidity because these variables were not systematically recorded; this is addressed in the Strengths and Limitations section.

Complete blood count parameters used for the calculation of inflammatory indices were obtained from the last routine preoperative blood sample collected within seven days before surgery at the preoperative assessment performed once the decision to operate had been taken. Complete blood count parameters were measured on a Sysmex XN-1000 automated hematology analyzer (Sysmex Corporation, Kobe, Japan). One further patient was excluded from the inflammatory feature sets because the differential leukocyte count was incomplete, leaving 224 patients with 59 high-grade events for those analyses.

Estrogen and progesterone receptor status was determined on the hysterectomy specimen; receptor immunohistochemistry is not performed on preoperative biopsy or curettage material at the study center. These variables were therefore not available at the intended time of prediction and were not used as predictors in any model. The preoperative variables eligible for model development are given in Table 1; these receptor variables are reported in Table 2 as descriptive characteristics.

The study protocol was approved by the Non-Interventional Clinical Research Ethics Committee of Sivas Cumhuriyet University (decision No. 2024/09-50, dated 19 September 2024), and this study was conducted in accordance with the principles of the Declaration of Helsinki. This study was approved as a retrospective review of hospital records; the requirement for written informed consent was waived on that basis, and the arrangements for confidentiality and pseudonymization are stated in the Informed Consent Statement; the requirement for written informed consent was waived on that basis, and the arrangements for confidentiality and pseudonymization are stated in the Informed Consent Statement. The approved protocol covered all patients who underwent surgery for endometrial cancer at the study center during the study period; the reference to endometrioid histology in the protocol title denotes the predominant subtype and was not applied as an eligibility criterion. This study is reported in accordance with the STROBE recommendations for observational studies [17] and the TRIPOD + AI statement for prediction models developed with regression or machine learning methods [18].

**Table 3:** Systemic inflammatory indices by tumor grade. Effect size is the rank-biserial correlation; positive values indicate higher levels in the high-grade group. AUC values are direction-corrected. q values were obtained by Benjamini–Hochberg correction within the family of nine indices.

Index	Low-Grade Median (IQR)	High-Grade Median (IQR)	Effect Size (Rank-Biserial r)	AUC (95% CI)	*p*	q (BH–FDR)
NLR	2.87 (1.77–4.81)	2.64 (2.01–4.81)	−0.010	0.505 (0.420–0.590)	0.906	1.000
dNLR	2.12 (1.40–3.39)	1.99 (1.55–3.37)	−0.040	0.520 (0.435–0.605)	0.650	1.000
PLR	150.11 (115.55–212.99)	154.12 (121.86–271.14)	+0.067	0.533 (0.446–0.621)	0.449	1.000
MLR	0.23 (0.17–0.36)	0.24 (0.19–0.38)	+0.075	0.537 (0.452–0.622)	0.394	1.000
LMR	4.34 (2.75–5.95)	4.11 (2.65–5.28)	−0.069	0.535 (0.449–0.620)	0.432	1.000
SII	808.62 (505.43–1367.93)	701.06 (500.35–1781.97)	+0.000	0.500 (0.412–0.588)	1.000	1.000
SIRI	1.28 (0.72–2.84)	1.20 (0.80–2.61)	+0.004	0.502 (0.418–0.586)	0.962	1.000
PIV	350.12 (189.92–740.65)	346.15 (188.28–992.59)	+0.028	0.514 (0.428–0.600)	0.749	1.000
ELR	0.04 (0.02–0.08)	0.07 (0.04–0.10)	+0.252	0.626 (0.545–0.707)	0.004	0.036

Statistical tests. Group medians were compared with the Mann–Whitney U test, the distribution of every index departing from normality (Shapiro–Wilk), and effect sizes are reported as the rank-biserial correlation. Confidence intervals for the area under the curve were computed by the DeLong method. The false discovery rate was controlled across the family of inflammatory indices by the Benjamini–Hochberg procedure; q denotes the adjusted *p* value. All tests were two-sided at α = 0.05. Analyses were performed in Python 3.13.15 (scikit-learn 1.6.1, numpy 2.1.3, scipy 1.16.3, statsmodels 0.14.6).

### 2.2. Data Preprocessing and Quality Control

Patient identifiers were removed and replaced with sequential study numbers; the data were therefore pseudonymized rather than anonymized, and were handled under a signed confidentiality undertaking. Two data-quality problems were identified in the complete blood count. First, one patient had a white blood cell (WBC) count recorded as 1324 × 10^3^/µL, an evident decimal-shift error; the value was corrected to 13.24 × 10^3^/µL, which was independently confirmed by the differential sum of that patient (13.23 × 10^3^/µL). Second, in four patients, the absolute difference between the recorded WBC and the sum of the differential (neutrophils + lymphocytes + monocytes + eosinophils) exceeded 2 × 10^3^/µL. Because the cohort-wide median of this difference was 0.03 × 10^3^/µL (basophil range), and because the neutrophil percentage computed against the differential sum fell within the cohort distribution in these patients, whereas the percentage computed against the recorded WBC did not, the WBC field rather than the differential components was judged erroneous, and WBC was recomputed as the differential sum. This is not an imputation for derived NLR (dNLR) since dNLR ≡ N/(WBC − N) and WBC − N = L + M + E + basophils; the computation therefore reproduces the definition excluding basophils. This correction was applied before the collinearity diagnostics reported in Section 2.4. Missing data were handled by complete-case analysis within each analytic block, with the corresponding n reported for every comparison. The number and proportion of missing values for each candidate predictor, and the joint missingness pattern, are given in Supplementary Table S2a,b; included and excluded patients are compared in Supplementary Table S2c. As a sensitivity analysis, the clinical and combined models were also fitted on all patients using multiple imputation by chained equations, with the imputation model fitted inside each training fold so that held-out patients contributed nothing to imputation.

### 2.3. Calculation of Inflammatory Indices

Nine indices were computed from the preoperative complete blood count: NLR = N/L; dNLR = N/(WBC − N); PLR = P/L; MLR = M/L; LMR = L/M; SII = P × N/L [19]; SIRI = N × M/L; PIV = N × P × M/L [20]; and ELR = E/L, where N, L, M, E and P denote absolute neutrophil, lymphocyte, monocyte, eosinophil and platelet counts (×10^3^/µL). The prognostic nutritional index was not included: it incorporates serum albumin rather than being derived solely from the differential count, and albumin was missing in 44 patients, which would have reduced the complete-case sample for every panel containing it.

### 2.4. Multicollinearity Diagnostics and Feature Sets

Because all indices are algebraic functions of the same five cell counts (e.g., SII = PLR × NLR; PIV = SII × M; SIRI = NLR × M), severe multicollinearity was anticipated and formally assessed with variance inflation factors (VIFs) and the condition number (κ) of the standardized design matrix. In the full 14-variable panel (NLR, dNLR, PLR, MLR, SII, SIRI, PIV and ELR, together with the white blood cell count and the neutrophil, lymphocyte, monocyte, eosinophil and platelet counts), the maximum VIF was 660.4 and κ was 110.3. Reduction proceeded in two stages: mathematically redundant variables were first removed by inspection (MLR, the reciprocal of LMR, Spearman’s |ρ| = 1.000, LMR being retained in its place; dNLR, algebraically redundant with NLR, both being ratios of the neutrophil count to a lymphocyte-derived denominator; WBC, Spearman’s ρ = 0.952 with neutrophils; eosinophils, Spearman’s ρ = 0.932 with ELR), followed by greedy elimination until no VIF exceeded 10 while protecting NLR. The reduced panel had a maximum VIF of 8.36 and κ of 7.2.

The variance inflation factor computation and the subsequent greedy elimination were performed once on the full design matrix, before cross-validation. Because these quantities are computed from the design matrix alone and are independent of the outcome, no information about the grade of held-out patients enters the definition of the feature sets; unsupervised screening of this kind is generally accepted as not invalidating subsequent cross-validation [21]. The reduction was nonetheless repeated independently within each outer training fold, and the retention frequency of each variable is reported in Supplementary Table S4.

Four feature sets were compared. Set A comprised the reduced inflammatory panel (neutrophils, lymphocytes, monocytes, platelets, NLR, LMR, PIV, ELR). Set A-full comprised the original 14-variable panel and was retained only for sensitivity analysis. Set B comprised preoperative clinical variables (age, CA-125 and preoperative serum albumin). Set C combined Sets A and B. Estrogen and progesterone receptor status were excluded for the reasons given in Section 2.1. With three candidate predictors and 48 events, the events-per-variable ratio for Set B is 16.0.

Deliberate exclusions: Histologic subtype was excluded from all models. Serous and clear-cell carcinomas are classified as high-grade by definition in the WHO 2020 system; using subtype as a predictor of grade therefore constitutes tautological prediction and artificially inflates apparent model performance. In a post hoc check using the prespecified model under an otherwise identical procedure, adding histologic subtype to the preoperative clinical set raised apparent discrimination from AUC 0.684 (95% CI 0.588–0.778) to 0.772 (0.669–0.869); both values arise from that check and are not directly comparable with Table 4. Variables that are consequences of grade rather than antecedents—FIGO stage, lymph node metastasis, omental involvement, postoperative albumin, and length of stay—were likewise excluded to prevent outcome leakage.

Because serous, clear-cell and undifferentiated carcinomas are high-grade by definition and 92.9% of non-endometrioid tumors in this cohort were G3, excluding subtype as a predictor does not by itself remove its influence: predictors correlated with subtype may carry the same information indirectly. All analyses were therefore repeated in three cohorts—the full cohort, endometrioid tumors only, and the subgroup in whom hormone receptor status had been assessed—and the distribution of subtype by grade is reported in Supplementary Table S5a.

### 2.5. Statistical Analysis

Normality was assessed within each group by the Shapiro–Wilk test and variance homogeneity by the Levene test. Continuous variables were compared with the Student or Welch *t* test when normality held in both groups (effect size: Hedges’ g) and with the Mann–Whitney U test otherwise (effect size: rank-biserial correlation). Categorical variables were compared with the Pearson chi-square test, or Fisher’s exact test when any expected cell frequency fell below five (effect size: Cramér’s V). The false discovery rate was controlled within the family of nine inflammatory indices using the Benjamini–Hochberg procedure [22]. Receiver operating characteristic analysis was performed with 95% confidence intervals computed by the DeLong method [23]; optimal cut-off points were derived from the Youden J index and sensitivity/specificity intervals from the Wilson score method. Two-sided α was 0.05. Analyses were performed in Python 3.13.15 (numpy 2.1.3, scipy 1.16.3, scikit-learn 1.6.1, statsmodels 0.14.6, shap 0.52.0).

Because the primary hypothesis of this study is one of absence, non-significance was not treated as evidence of no effect. A non-superiority test against a discrimination margin was therefore performed. This is not an equivalence test: a single one-sided null hypothesis is tested, namely that the area under the curve is at least 0.70, against the alternative that it is lower. The bootstrap is conditional on fixed out-of-fold predictions and therefore does not incorporate model-refitting variability, and this analysis is accordingly reported as an exploratory sensitivity analysis rather than as a confirmatory one. For each inflammatory index and for each model, the null hypothesis that the area under the curve is at least 0.70 was tested against the one-sided alternative that it is lower; a margin of 0.70 was adopted post hoc as the conventional lower bound of acceptable discrimination [24]. The incremental contribution of the inflammatory indices was assessed by the paired difference in area under the curve between the combined and the clinical model, restricted to patients with predictions from both models, against a margin of +0.05. One-sided 95% upper confidence bounds and *p* values were obtained from 5000 stratified bootstrap resamples of the out-of-fold predictions, with identical resampling indices applied to both models in the paired comparison; the paired result was cross-checked against the DeLong covariance estimate. The claim that no index in the family reaches the margin constitutes an intersection-union test, in which rejection requires every component test to reject and the type I error rate is therefore controlled at the nominal level without adjustment for multiplicity [25]; no correction was consequently applied to the non-superiority tests, in contrast to the Benjamini–Hochberg procedure used for the primary comparisons.

The primary analysis uses a single prespecified algorithm rather than selection among several. Elastic-net logistic regression was chosen in advance for reasons specific to this dataset: the events-per-variable ratio ranges from 1.4 to 16.0 across feature sets and cohorts and is below ten for every set other than the preoperative clinical set in the full cohort; flexible algorithms require substantially more events than penalized regression to reach comparable performance [26]; and models developed from small samples by flexible methods are unstable in the sense that individual predicted risks vary considerably across samples of the same size [27]. A shrinkage-based linear model is the appropriate default in this regime and removes the data-driven choice among algorithms as a source of optimism. An elastic-net logistic regression was tuned over a two-dimensional grid of penalty strength and mixing parameter; the configuration retained was that with the best inner-loop score, and the one-standard-error rule was examined separately as a sensitivity analysis (Section 3.6). Performance was estimated by five-fold outer cross-validation repeated twenty times with out-of-fold probabilities averaged over repetitions. The comparison among nine classifiers is retained as an exploratory analysis.

### 2.6. Machine Learning

Nine classifiers were evaluated: elastic-net logistic regression, radial-basis support vector machine, k-nearest neighbors, random forest, extremely randomized trees, XGBoost, LightGBM, CatBoost, and gradient boosting. The primary analysis used repeated nested cross-validation: a stratified five-fold outer loop for performance estimation and a stratified five-fold inner loop for hyperparameter search, repeated twenty times with out-of-fold probabilities averaged across repetitions. Imputation, standardization and hyperparameter selection were performed within each training fold and applied to the corresponding outer test fold. The classification threshold was likewise determined by the Youden index within each training fold and applied to the corresponding outer test fold; it was not selected after pooling out-of-fold predictions. The complete hyperparameter specification, including the search grid, the number of configurations evaluated and the software versions used, is given in Supplementary Table S6. Performance was computed from out-of-fold predicted probabilities pooled across outer folds, and 95% confidence intervals were obtained from 5000 stratified bootstrap resamples. Class imbalance was addressed with class weighting rather than synthetic oversampling. Given the imbalance (26.2% positive), the area under the precision–recall curve, balanced accuracy and the Matthews correlation coefficient are reported alongside AUC-ROC; raw accuracy is not since a model predicting the majority class for every patient attains 73.8%. Calibration was assessed by flexible calibration curves obtained by locally weighted scatterplot smoothing of the observed outcome against pooled out-of-fold predicted probability, together with the calibration intercept, the calibration slope and the Brier score [28]. Quantile binning was not used: with 17 to 48 events depending on the cohort, decile bins would contain four or fewer events and the resulting curve would be dominated by sampling noise. The Hosmer–Lemeshow test is not reported since its power is low at this sample size and its result depends on an arbitrary grouping. Because class weighting inflates predicted probabilities systematically, and because net benefit is defined on the absolute probability scale, decision curve analysis [29] was performed on probabilities recalibrated by logistic recalibration of the out-of-fold predictions, and net benefit is reported with bootstrap confidence bands.

To establish the performance attainable by chance in this design, the outcome labels were randomly permuted and the full modeling pipeline re-run; the 95th percentile of the resulting null AUC distribution is reported. Post hoc sample size requirements for detecting a given AUC against the null of 0.50 were computed with the Hanley–McNeil variance approximation [30].

The exploratory comparison among nine classifiers is reported in Section 3.6 and Supplementary Table S5b. In that analysis, the algorithm with the highest pooled out-of-fold AUC was identified for each feature set, which uses outer-fold performance and therefore carries selection optimism; the maximum across algorithms is an optimistically biased estimate of the performance of any one of them, and it is presented on that understanding.

The decision the model is intended to inform is whether to proceed to comprehensive surgical staging with systematic pelvic and para-aortic lymphadenectomy, or to refer to a gynecologic oncology center, in a patient whose preoperative biopsy grade is low or indeterminate; the staging decision and the risk groups it rests on follow current European guidance [31]. At a threshold probability p, decision curve analysis implies an exchange rate of p/(1−p): a clinician acting at *p* = 0.05 accepts nineteen unnecessary staging procedures for each additional high-grade tumor identified, at *p* = 0.10 nine, at *p* = 0.20 four and at *p* = 0.40 approximately 1.5. The range 0.05 to 0.40 was chosen to span the clinically defensible exchange rates, with the upper bound above the base rate of high-grade disease in the cohort, and the results are reported only within it [32].

### 2.7. Explainability Analysis

For explanatory purposes, the best-performing tree-based model of the exploratory analysis in each feature set was refitted on the complete dataset and interpreted using TreeSHAP [33]. Because SHAP attributions are unstable in the presence of correlated features, feature rankings were cross-checked against permutation importance and the agreement between the two rankings quantified by the Spearman correlation. SHAP values were computed with the shap package (version 0.52.0) using TreeExplainer with model_output = “raw”; the values are therefore on the log-odds scale and not on the probability scale. They quantify each feature’s contribution to the output of the fitted model for a given input and are properties of that model rather than of the underlying biology: they do not identify causal effects and do not correspond to adjusted associations of the kind a regression coefficient would express. SHAP values were computed for explanatory purposes only; performance estimates throughout are those from nested cross-validation. Standardized coefficients of the prespecified elastic-net model are reported alongside them.

### 2.8. Use of Generative Artificial Intelligence

During the preparation of this manuscript, the authors used Claude Opus 4.1 (Anthropic PBC, San Francisco, CA, USA; accessed June–August 2026) for language editing, sentence-structure correction, and assistance with manuscript formatting and reference management. Artificial intelligence assistance was also used in debugging the Python analysis scripts; all code was reviewed, verified, and executed by the authors. All analyses reported in this article—data preprocessing, multicollinearity diagnostics, statistical testing, nested cross-validation, non-superiority testing, calibration and decision curve analysis, and SHAP computation—were designed and executed by the authors in Python 3.13.15 (numpy 2.1.3, scipy 1.16.3, scikit-learn 1.6.1, statsmodels 0.14.6, shap 0.52.0). No generative artificial intelligence tool was used for study design, data collection, data analysis, or the interpretation of the results, and no numerical result, table, or figure reported in this article was generated by such a tool. The authors reviewed and edited all output and are fully responsible for the content of this publication.

## 3. Results

### 3.1. Patient Characteristics

Patients with high-grade tumors were significantly older (65.1 ± 10.3 versus 57.5 ± 9.7 years; *p* < 0.001; Hedges’ g = 0.78). Hormone receptor status had been assessed in 159 of 225 patients, and every assessed tumor was receptor-positive; the cohort therefore contains no receptor-negative tumors. Receptor status had not been assessed in 37 patients and no record was available in a further 29. Assessment was less frequent in high-grade and in non-endometrioid tumors (Supplementary Table S7), and no significance test is reported for this variable since such a test would compare grade with whether the assay was requested rather than with receptor status. Preoperative albumin was marginally lower in high-grade disease (*p* = 0.035), whereas the difference in postoperative albumin was pronounced (*p* < 0.001). CA-125 did not differ significantly (*p* = 0.087). Preoperative characteristics eligible for model development are summarized in Table 1 and postoperative or pathological characteristics, reported for description only, in Table 2.

### 3.2. Inflammatory Indices

None of NLR, dNLR, PLR, MLR, LMR, SII, SIRI or PIV differed significantly between grade groups, with rank-biserial effect sizes below 0.08 in absolute value and areas under the curve between 0.50 and 0.54. The difference for SII was exactly null (*p* = 1.000). Only ELR retained statistical significance after false discovery rate correction (q = 0.036). Its discrimination was nonetheless modest—AUC 0.626 (95% CI 0.545–0.707), with a lower confidence bound close to chance—and the one-sided non-superiority bound of 0.694 lies close to the margin of 0.700, so that even the exclusion of clinically useful discrimination is marginal for this index. The associated cut-off was derived from the same cohort by the Youden index and is therefore optimism-prone. ELR is accordingly interpreted as an exploratory hematological index showing a statistical association with grade in this cohort, not as an independent or clinically useful predictive biomarker. The results are given in Table 3.

### 3.3. Model Performance

**Figure 2:**
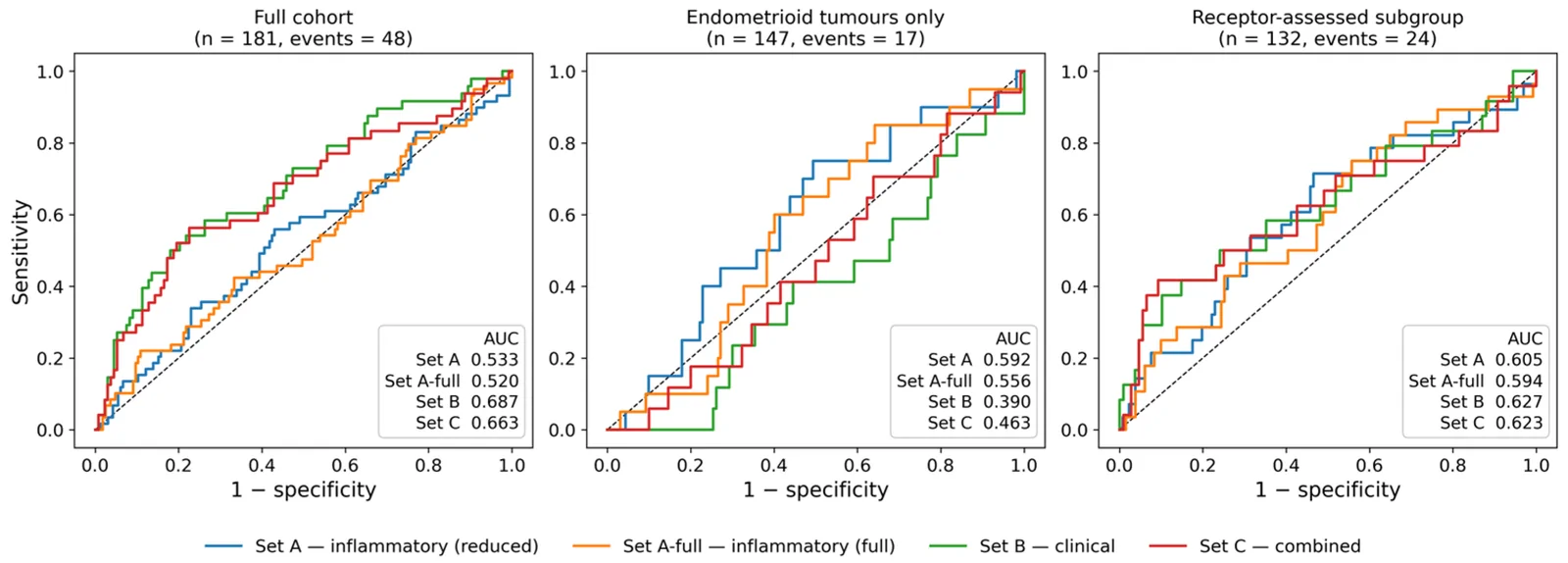
Out-of-fold receiver operating characteristic curves for the prespecified elastic-net model in each feature set, shown separately for the three cohorts. The dashed diagonal represents chance-level discrimination (AUC = 0.50). The number of patients contributing to each feature set is given in the legend of each panel (Table 4).

Performance of the prespecified model within each feature set and cohort is summarized in Table 4. In the full cohort, the reduced inflammatory panel reached AUC 0.533 (95% CI 0.443–0.621) and the full panel 0.520 (0.432–0.609), both with intervals spanning chance level. Preoperative clinical variables reached 0.687 (0.590–0.772) and the combined set 0.663 (0.562–0.752). The corresponding out-of-fold receiver operating characteristic curves are shown in Figure 2.

The three-cohort structure changes the interpretation of these figures. Of 59 high-grade events, 39 (66.1%) occurred in non-endometrioid tumors, and 92.9% of non-endometrioid tumors were G3 (Supplementary Table S5a). Within endometrioid tumors, the preoperative clinical set reached AUC 0.390 (0.257–0.524) and within the receptor-assessed subgroup, it reached 0.627 (0.485–0.765); in both cohorts, the confidence interval includes 0.50. The discrimination observed for clinical variables in the full cohort therefore does not survive restriction to a histologically homogeneous population.

Permutation of the outcome labels with the full modeling pipeline gave a null distribution with a mean of 0.499 and a 95th percentile of 0.586 for the inflammatory set against an observed 0.533 (permutation *p* = 0.254), and a mean of 0.495 with a 95th percentile of 0.587 for the clinical set against an observed 0.687 (*p* = 0.005). A post hoc calculation indicated that approximately 140 events, in around 534 patients at the case-to-control ratio of this cohort, would be required to distinguish an AUC of 0.58 from 0.50 with 80% power; the present cohort contains 59.

#### Incremental Value of the Inflammatory Indices

The contribution of the inflammatory panel over the preoperative clinical variables was assessed within the common complete-case population of each cohort on four scales. In the full cohort (n = 181, 48 events), the paired difference in area under the curve between the combined and clinical models was −0.025 (95% CI −0.062 to +0.012; two-sided *p* = 0.212), the DeLong test gave *p* = 0.190, the difference in Brier score was +0.005 and the difference in area under the precision–recall curve was −0.022. The one-sided upper bound of the paired difference was +0.007, which excludes the prespecified margin of +0.05. In the endometrioid cohort, the paired difference was +0.074 (−0.097 to +0.243; *p* = 0.405), and in the receptor-assessed cohort, it was −0.004 (−0.103 to +0.092; *p* = 0.942).

The negative point estimate in the full cohort does not establish that inflammatory variables reduce predictive performance: the confidence interval includes zero and the two-sided tests are not significant in any cohort. What the analysis supports is the narrower conclusion that any incremental contribution is bounded below the smallest difference considered clinically relevant.

### 3.4. Explainability

**Figure 3:**
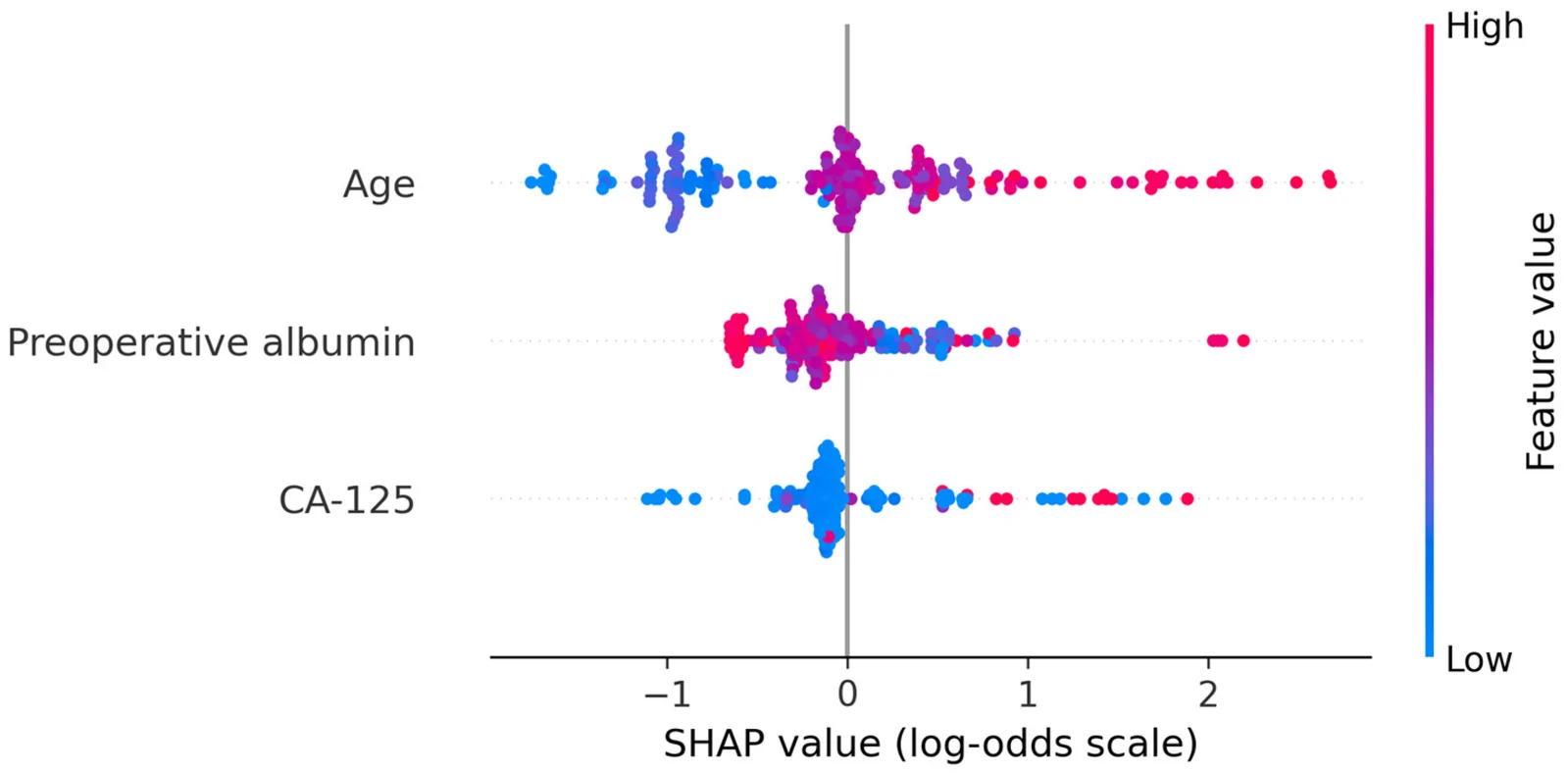
SHAP beeswarm plot for the exploratory tree model fitted to the preoperative clinical set. Values are on the log-odds scale. Each point represents one patient; color encodes the feature value and the horizontal position the contribution to the model output.

In the exploratory tree model fitted to the preoperative clinical set, the largest mean absolute SHAP values, on the log-odds scale, were those of age (0.668), preoperative albumin (0.338) and CA-125 (0.315) (Figure 3). In the prespecified elastic-net model, the standardized coefficients were age +0.612, CA-125 +0.248 and preoperative albumin −0.086. These quantities describe how each model uses its inputs; they do not establish biological causality or independent prognostic effects, and they are not measures of discrimination. In the inflammatory model, ELR had the largest mean absolute SHAP value; this ranking describes a model whose discrimination is not distinguishable from chance and should not be interpreted as evidence of biological relevance. Feature attributions are given in Table 5.

### 3.5. Calibration and Clinical Utility

**Figure 4:**
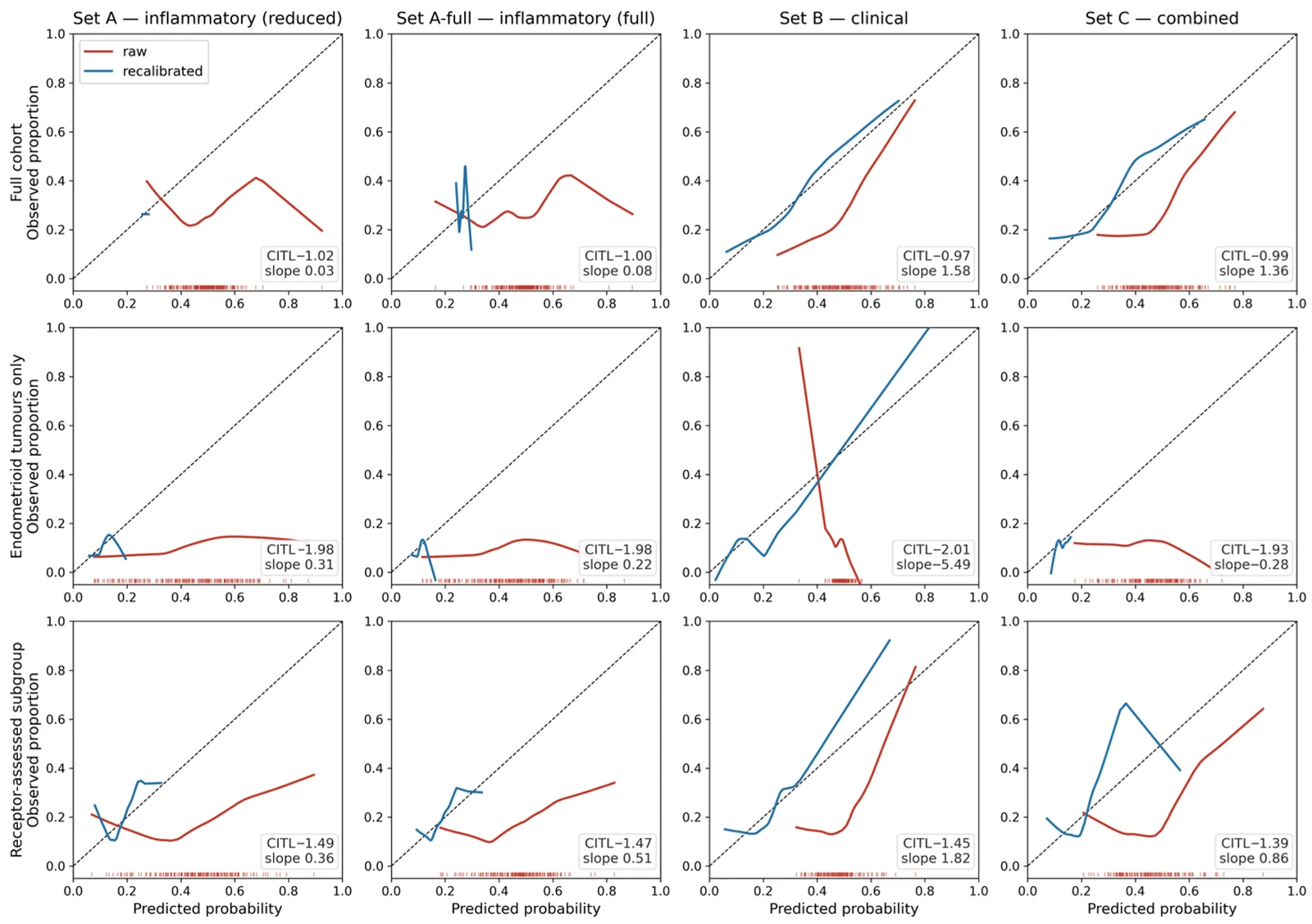
Flexible calibration curves for the prespecified elastic-net model. Rows correspond to the three cohorts and columns to the four feature sets. Red curves show the raw predicted probabilities, blue curves the probabilities after logistic recalibration, and the dashed diagonal perfect calibration. The red tick marks along the horizontal axis show the distribution of raw predicted probabilities. CITL, calibration-in-the-large (calibration intercept); slope, calibration slope. Both values are reported for the raw probabilities; after recalibration they are 0 and 1 by construction, so the recalibrated curves display only residual non-linear miscalibration.

The raw predicted probabilities were systematically miscalibrated. Calibration-in-the-large was −1.02 in the full cohort, −1.98 in the endometrioid cohort and −1.49 in the receptor-assessed cohort; in each case, this corresponds closely to the log-odds of the outcome prevalence in that cohort, identifying class weighting as the cause. Discrimination is unaffected because the ranking of patients is preserved, but the Brier score is not: in the full cohort, it was 0.225 for the clinical set against 0.195 for a prevalence-only prediction. After logistic recalibration, the Brier score fell below the prevalence-only value in every cohort and feature set—0.177 against 0.195 for the clinical set—and calibration intercept and slope were 0 and 1 by construction (Figure 4).

**Figure 5:**
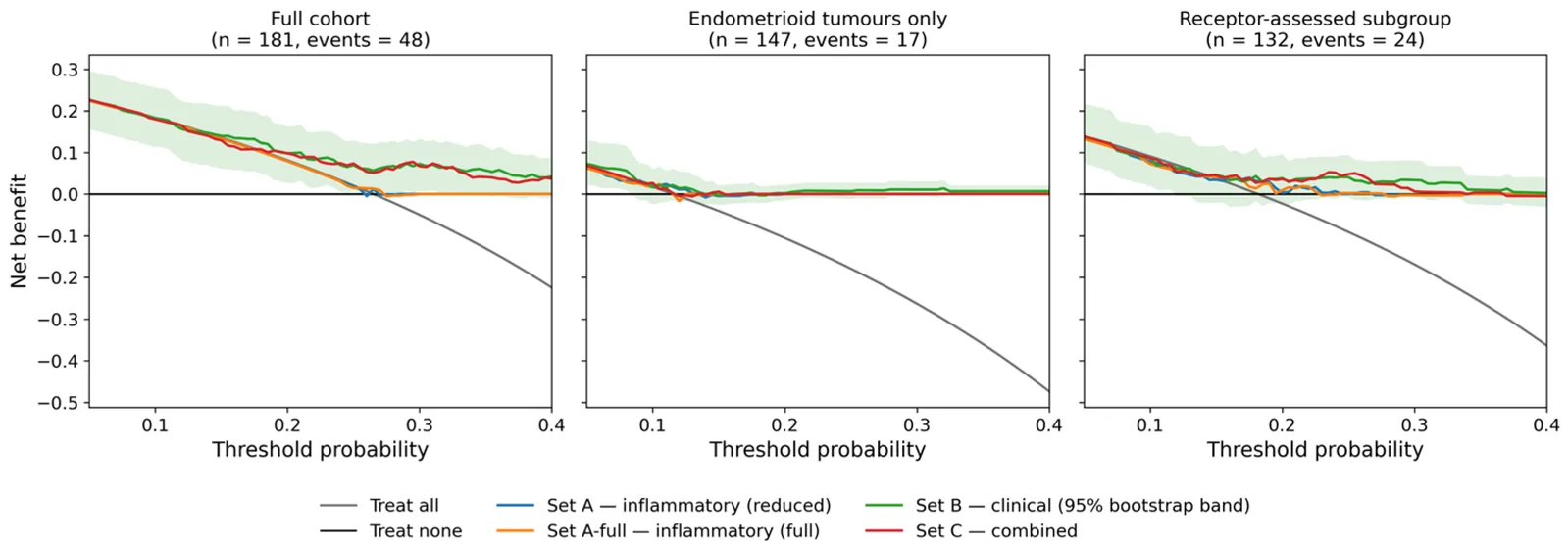
Decision curve analysis on recalibrated out-of-fold probabilities, shown separately for the full cohort, endometrioid tumors and the receptor-assessed subgroup. The shaded green band is the 95% bootstrap confidence band for the preoperative clinical set, shown for that set alone so that the four curves remain distinguishable. The grey line represents the treat-all strategy and the horizontal black line the treat-none strategy; the threshold range shown is 0.05 to 0.40. The number of patients contributing to each feature set is as in Figure 2 and Table 4.

Decision curve analysis was therefore performed on the recalibrated probabilities (Figure 5). Below the prevalence of high-grade disease in each cohort—the range in which treat-all is the relevant comparator—no model exceeded treat-all. In the full cohort, the clinical model attained a net benefit of 0.183 at a threshold of 0.10 against 0.183 for treat-all (difference −0.001, 95% CI −0.014 to +0.008) and 0.098 at 0.20 against 0.082 (difference +0.017, −0.025 to +0.054). Above the prevalence, treat-all becomes negative and treat-none is the relevant comparator; the clinical model retained a small positive net benefit in the full cohort (0.073 at a threshold of 0.30) but essentially none within endometrioid tumors (0.011 at the same threshold). Discrimination and clinical utility are distinct properties: a model can rank patients better than chance and still add nothing over the strategies available without it, and that is the case here at every threshold at which treat-all is a live option.

### 3.6. Sensitivity Analyses

Six sensitivity analyses were performed. First, complete exclusion of the four patients with inconsistent blood-count entries left all findings unchanged (for example, ELR: AUC 0.627 versus 0.626 in the primary analysis). Second, an alternative dichotomization (G1 versus G2–G3) was examined; despite an increase in the number of events from 59 to 92 and improved class balance, no inflammatory index survived false discovery rate correction and the performance of the inflammatory set decreased, indicating that what limited signal exists is specific to G3 tumors. Third, the collinearity reduction was repeated independently within each outer training fold; lymphocytes, monocytes, neutrophils, platelets, NLR, ELR and LMR were retained in every one of 25 folds and PIV in 64%, reproducing the published Set A (Supplementary Table S4). Fourth, all feature sets were evaluated in the common complete-case population of each cohort (Supplementary Table S3). Fifth, the clinical and combined models were refitted on all patients using multiple imputation by chained equations within the cross-validation folds, giving AUC 0.693 (95% CI 0.608–0.774) in the full cohort against 0.687 for the complete-case analysis, and 0.397 against 0.390 in the endometrioid cohort (Supplementary Table S10). Sixth, the effect of the hyperparameter selection rule was examined: under the one-standard-error rule, the penalty parameter was selected at the lower bound of the search grid in 52% to 88% of folds, shrinking the coefficients almost to zero and reducing the AUC of the clinical set in that comparison from 0.690 to 0.642 while raising its Brier score from 0.224 to 0.248 (Supplementary Table S9). The primary analysis therefore refits on the best inner-loop score.

**Figure 6:**
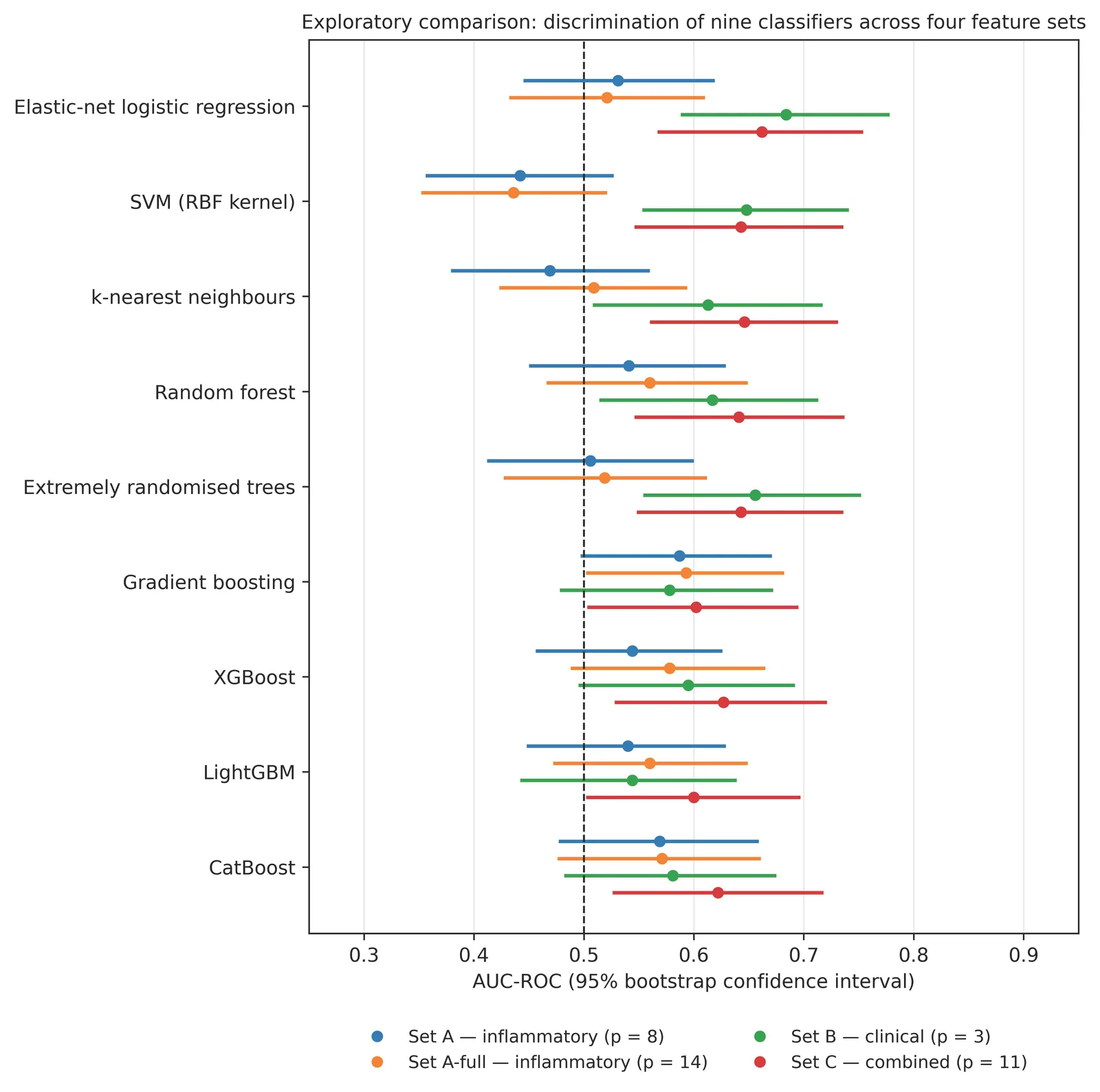
Exploratory comparison: discrimination of nine classifiers across four feature sets in the full cohort, shown as AUC with 95% bootstrap confidence intervals. The dashed line marks chance level (AUC = 0.50). Seventeen of the eighteen intervals in the two inflammatory sets include 0.50. In the preoperative clinical set, the best of the nine algorithms is the prespecified elastic net itself (AUC 0.684 against 0.687 for the primary analysis). Because the algorithm shown for each feature set would be selected on out-of-fold performance, these values carry selection optimism and are reported as exploratory; numerical values are given in Supplementary Table S5b.

Non-superiority testing gave the following results: Against a margin of 0.70, the one-sided 95% upper confidence bound fell below the margin for every inflammatory index and for both inflammatory models: 0.575 for SIRI, 0.577 for SII and for NLR, 0.591 for dNLR, 0.590 for PIV, 0.607 for LMR, 0.609 for PLR, 0.611 for MLR, 0.694 for ELR, 0.607 for the reduced panel, and 0.595 for the full panel (Supplementary Table S8). Since rejection of the family null requires every component test to reject, the null that some index in the family attains an area under the curve of 0.70 is rejected. The bound for ELR lies 0.006 below the margin; so, the family-level conclusion would not survive a materially lower margin. The incremental contribution of the inflammatory indices is reported in Section Incremental Value of the Inflammatory Indices. An exploratory comparison of nine classifiers across the four feature sets is shown in Figure 6.

## 4. Discussion

In this cohort of 225 women with endometrial cancer, most systemic inflammatory indices did not demonstrate clinically useful discrimination between high-grade and low-grade tumors. Preoperative clinical variables achieved moderate discrimination in the full cohort, but that discrimination did not survive restriction to endometrioid histology, and adding inflammatory indices to them did not provide incremental value beyond the prespecified margin. The model presented here is not intended to replace preoperative endometrial sampling, which remains the clinical standard and the relevant comparator, but to ask whether preoperatively available blood and clinical data add anything to it. The finding was consistent across four independent analytic approaches: univariate testing with false discovery rate correction, receiver operating characteristic analysis, multivariable machine learning with nested validation, and sensitivity analysis under an alternative grade dichotomization. Routinely available clinical variables achieved moderate discrimination, but adding inflammatory indices to them did not improve performance.

Our results contrast with reports describing NLR, PLR and MLR as informative in endometrial and other gynecological cancers [6,7]. Several methodological differences may account for this divergence. Many published analyses evaluate multiple indices without correction for multiple comparisons; in the present data, eight of nine indices lost significance after Benjamini–Hochberg correction, and the single surviving index reached an AUC of only 0.626. Multicollinearity among these indices is rarely addressed, although they are algebraic transformations of the same five cell counts; variance inflation factors reached 660 in the unreduced panel here. Finally, the distinction between discriminating malignant from benign disease and discriminating grade within malignancy is often blurred. Systemic inflammation may well accompany the presence of a tumor without tracking its differentiation, and our negative result for grade is not in conflict with positive reports for the case–control question.

This distinction between prognosis and grade is supported by the recent literature. Meta-analyses in endometrial and gynecological cancer report consistent associations between the systemic immune-inflammation index and survival endpoints, with pooled hazard ratios around two for overall and progression-free survival [8,9], and single-center series report similar associations for the neutrophil-to-lymphocyte and platelet-to-lymphocyte ratios [10]. These are prognostic questions, defined over time-to-event outcomes in patients whose disease burden is already established. Grade is a static histopathological property assigned at diagnosis, and the evidence that inflammatory indices track it is far weaker: a 2025 series of low-risk endometrial cancer found no association between the neutrophil-to-lymphocyte ratio and recurrence-free survival, whereas tissue-level immune scores were associated with outcome [11], and a prospective multicentric study of 163 women with FIGO 2023 stage I disease reported associations of the neutrophil-, monocyte- and platelet-to-lymphocyte ratios with depth of myometrial infiltration at borderline significance levels [12]. Our result is therefore not in conflict with the prognostic literature; it delimits it. Systemic inflammation may accompany tumor burden without indexing tumor differentiation.

A third question must also be separated from ours. Several studies ask whether inflammatory indices distinguish endometrial malignancy from benign uterine disease, and there the indices perform reasonably: a study of 80 cancers and 74 benign controls reported areas under the curve of 0.777, 0.675 and 0.698 for the neutrophil-, platelet- and monocyte-to-lymphocyte ratios, rising to 0.780 in combination [7]. That is a diagnostic contrast between diseased and non-diseased uteri, not a gradation within a single malignancy, and two features of such designs invite caution. The reported thresholds are unstable across the literature: optimal cut-offs for the neutrophil-to-lymphocyte ratio in endometrial cancer have been placed anywhere between 2.02 and 4.68 across the studies reviewed in that report alone, each derived from the cohort in which it was applied. And case–control comparisons of this kind are vulnerable to confounding by age, which was roughly eight years higher in the malignant group of that study and was not adjusted for; age is itself the dominant clinical predictor in our data. Taken together, the literature supports a layered reading: inflammatory indices carry information about whether malignancy is present and about how a diagnosed patient will fare, but not about how the tumor is differentiated.

The sample size available for this question deserves emphasis beyond the usual statement of limitation. With 17 to 59 events and three to fourteen candidate predictors, the number of events per predictor ranged from 1.4 to 16.0 across feature sets and cohorts and was below the conventional threshold of ten in every set other than the preoperative clinical set in the full cohort; the formal criteria of Riley and colleagues [34,35] are more demanding still. A systematic review of prediction models developed with machine learning in oncology found that included studies fell short of the minimum required sample size by a median of around three hundred events, and that a sample size justification was reported in only one of thirty-six studies [36]. Simulation work has shown that flexible algorithms require substantially more events than logistic regression to reach comparable performance [26]. The practical consequence is instability: the model that emerges from a small development sample is one of many that could have emerged from a different sample of the same size, and an individual predicted risk may vary considerably across that set [27]. We observed this directly. When the modeling was repeated, the algorithm selected as best differed across feature sets and across repetitions, even though the corresponding areas under the curve were close and their confidence intervals overlapped extensively. A single best model reported without this instability would overstate the strength of the evidence.

The exclusion of histologic subtype merits emphasis. Because serous and clear-cell carcinomas are high-grade by definition, adding subtype as a predictor of grade raised the apparent discrimination of the prespecified clinical model from AUC 0.684 to 0.772 in a post hoc check (Section 2.4). Reported model performance in comparable studies should be interpreted with this circularity in mind.

The distribution of histologic subtypes deserves emphasis because it accounts for a substantial part of what the full-cohort analysis appears to show. Of 59 high-grade events, 39 occurred in non-endometrioid tumors, and 92.9% of non-endometrioid tumors were high-grade. Serous, clear-cell and undifferentiated carcinomas are high-grade by definition; so, for two thirds of the events, the G3 versus G1–G2 contrast is a contrast between histologic subtypes rather than between grades within a single subtype. Excluding subtype as a predictor does not remove this influence because the remaining predictors carry the same information indirectly: mean age was 57.6 years in endometrioid and 67.4 years in non-endometrioid tumors, and among patients with a receptor record, receptor status had not been assessed in 12.0% of endometrioid against 47.4% of non-endometrioid tumors. Consistently with this, the preoperative clinical model fell from an area under the curve of 0.687 in the full cohort to 0.390 within endometrioid tumors. Reported model performance in mixed-histology cohorts should be interpreted with this in mind.

A further methodological point concerns explainability. SHAP attributions are computed independently of model performance; a model that does not discriminate will still yield a feature ranking. Reporting such a ranking as a finding—as the identification of an important biomarker—is not supported. We therefore report SHAP results for the inflammatory model together with the explicit statement that the underlying model is not distinguishable from chance, and we cross-validated all rankings against permutation importance.

This concern is not ours alone. A recent methodological appraisal argues that explainable artificial intelligence does not necessarily produce insights relevant to medical decision-making, that its output is difficult to keep free of causal reading, and that it can generate misplaced trust in a model or, conversely, unwarranted dismissal of one whose explanations conflict with clinical expectation; trust, on this account, should rest on validation and impact studies rather than on attribution plots [37]. Our data illustrate the first failure mode concretely. The inflammatory model yields a coherent, readable feature ranking headed by the eosinophil-to-lymphocyte ratio, and that ranking would be indistinguishable in presentation from one produced by a model that discriminates well. Only the accompanying performance estimate, and the permutation null against which it is compared, reveal that the ranking explains a model that is not distinguishable from chance. Feature attributions should therefore be reported downstream of discrimination, never in place of it.

Our own analysis illustrates this. During the analysis, a variable that had ranked high in the attribution plot of an earlier model specification was found, on returning to the source coding, not to record what its label described: the value we had read as a negative result denoted that the assay had not been requested, and non-assessment was concentrated in tumors that were already evidently non-endometrioid. The attribution was therefore to a data-collection artefact indexing histologic subtype, which is high-grade by definition, and the variable has been removed from every model for the separate reason that it is not available before surgery (Section 2.1). Nothing in the attribution plot indicated any of this: the ranking was coherent, stable across resampling, and consistent with clinical expectation. Feature attributions must therefore be reported downstream not only of discrimination but of data provenance, and never in place of either.

The clinical model, although statistically superior to the inflammatory models in the full cohort, provided no net benefit over the treat-all strategy at thresholds below the prevalence of high-grade disease once probabilities were recalibrated, and only a small advantage over treating no one above it—an advantage that disappeared within endometrioid tumors. Its discrimination likewise did not survive restriction to endometrioid histology. Grade is in any case established preoperatively in routine practice by endometrial biopsy, which performs considerably better than any model presented here. The clinical relevance of a preoperative grade-prediction model based on blood parameters is therefore limited even where moderate discrimination is observed. Discrimination and clinical utility are distinct quantities and should be reported together rather than one standing for the other [38].

### Strengths and Limitations

The strengths of this study include correction for multiple comparisons, explicit multicollinearity diagnostics, nested cross-validation with bootstrap confidence intervals, permutation-based null modeling, decision curve analysis on recalibrated probabilities, six sensitivity analyses, and the deliberate exclusion of tautological and outcome-derived predictors.

The principal limitations are the single-center retrospective design and the absence of external validation. With 59 events, this study is adequately powered to exclude clinically useful discrimination but not to exclude a small true effect: a post hoc calculation indicates that approximately 140 events, in around 534 patients at the case-to-control ratio of this cohort, would be required to detect an AUC of 0.58. Blood counts were obtained preoperatively without systematic exclusion of concomitant infection, corticosteroid use or inflammatory comorbidity, which may add noise. Body mass index and diabetes status, both of which influence inflammatory indices, were not available. Finally, the eosinophil-to-lymphocyte ratio finding is based on a single cohort with an optimism-prone cut-off derived from the same data and requires independent replication before any interpretation. Two features of the non-superiority analysis should be read with caution. The margin of 0.70 was adopted post hoc rather than registered in advance, and the result for ELR is close to it: an upper bound of 0.694 against a margin of 0.700. The family-level conclusion would therefore not survive a materially lower margin, and the bootstrap is conditional on fixed out-of-fold predictions and does not incorporate model-refitting variability; both analyses are reported as exploratory.

Hormone receptor status was determined on the hysterectomy specimen and was not available preoperatively; it was therefore excluded from all models.

The interval between blood sampling and surgery could not be quantified from the analytic dataset. Systemic inflammatory indices are time-sensitive, and heterogeneity in this interval would add noise to every index examined—a limitation that acts against the detection of an association rather than creating one.

Preoperative biopsy grade was not recorded in a form that could be extracted retrospectively; so, a direct comparison with the current clinical standard was not possible and the question of which patients are at risk of upgrading from biopsy to final pathology could not be addressed. That question is the more informative one clinically and is the appropriate design for prospective work.

Selection into the complete-case analysis was associated with CA-125 (*p* = 0.002), lymphocyte count (*p* < 0.001) and NLR (*p* = 0.043), but not with the proportion of high-grade disease (*p* = 0.99) or of endometrioid histology (*p* = 1.00), and a multiple-imputation analysis on all patients gave results close to the complete-case analysis.

Finally, a negative finding requires a different standard of evidence than a positive one. Non-significance alone establishes only absence of evidence; establishing evidence of absence requires that the effect be bounded away from a value considered relevant. We therefore tested non-superiority explicitly, against a margin of 0.70 for discrimination and of 0.05 for the incremental contribution of the inflammatory indices over the clinical variables. The claim that no index in the family reaches the margin is an intersection–union test, in which the null is rejected only if every component test rejects and the type I error rate is consequently preserved without adjustment for multiplicity [25]. What this design can exclude is discrimination of clinically useful magnitude; what it cannot exclude is a small true effect, for which a post hoc calculation indicates that approximately 140 events would be required. The appropriate reading of our result is therefore bounded: systemic inflammatory indices do not provide clinically useful discrimination of tumor grade in this setting, rather than that no association whatsoever exists.

## 5. Conclusions

Systemic inflammatory indices derived from routine complete blood counts did not demonstrate clinically useful discrimination between G3 and G1–G2 endometrial cancer in this cohort, and did not add incremental discriminative value beyond the prespecified margin over routinely available preoperative clinical variables. Age had the largest mean absolute SHAP value in the fitted clinical model, which nonetheless offered no advantage over the strategies available without it in decision curve analysis and lost its discrimination when the cohort was restricted to endometrioid histology. These results do not support the use of inflammatory indices for preoperative grade estimation in this setting; they bound the clinical usefulness of such indices rather than excluding any association between systemic inflammation and tumor grade. They also illustrate the importance of multiplicity control, multicollinearity diagnostics, nested validation, permutation-based null modeling, calibration assessment and attention to data provenance when applying machine learning and explainability methods to small clinical datasets.

## Figures and Tables

**Table 1:** Preoperative characteristics eligible for model development, by tumor grade. Continuous variables are given as mean ± SD when normally distributed and as median (interquartile range) otherwise.

Characteristic	Low-Grade (G1–G2) n = 166	High-Grade (G3) n = 59	*p*
Age (years), n = 166/59	57.46 ± 9.69	65.12 ± 10.26	<0.001 ***
CA-125 (U/mL), n = 162/58	16.28 (10.98–33.35)	20.30 (12.20–44.37)	0.087
Preoperative albumin (g/dL), n = 133/48	4.23 (3.97–4.46)	4.03 (3.62–4.36)	0.035 *

All variables in this table were eligible as candidate predictors in the preoperative models (Section 2.4). The inflammatory indices are reported separately in Table 3. Tests: Student’s t (age); Mann–Whitney U (CA-125, preoperative albumin). All tests two-sided. * *p* < 0.05; *** *p* < 0.001. Not corrected for multiple comparisons; correction was applied within the family of inflammatory indices (Table 3).

**Table 2:** Postoperative and pathological characteristics, by tumor grade, reported for description only.

Characteristic	Low-Grade (G1–G2)	High-Grade (G3)	*p*
Postoperative day-1 albumin (g/dL), n = 147/55	3.47 ± 0.44	3.11 ± 0.55	<0.001 ***
Length of stay (days), n = 155/54	8.00 (7.00–11.00)	12.00 (8.00–19.75)	<0.001 ***
Total lymph nodes harvested, n = 146/54	29.57 ± 10.91	29.89 ± 15.28	0.888
Pelvic lymph nodes harvested, n = 146/54	23.43 ± 8.35	21.17 ± 10.15	0.110
Para-aortic lymph nodes harvested, n = 146/54	5.00 (2.25–8.00)	8.50 (3.00–12.75)	0.014 *
Lymphadenectomy performed, n = 166/58	146 (88.0%)	54 (93.1%)	0.275
ER assessed and positive	131 (78.9%)	28 (47.5%)	—
ER not assessed	14 (8.4%)	23 (39.0%)	—
ER no record	21 (12.7%)	8 (13.6%)	—
PR assessed and positive	128 (77.1%)	27 (45.8%)	—
PR not assessed	17 (10.2%)	24 (40.7%)	—
PR no record	21 (12.7%)	8 (13.6%)	—

None of the variables in this table was eligible as a candidate predictor: postoperative albumin and length of stay, like FIGO stage, nodal metastasis and omental involvement (not shown), are consequences rather than antecedents of grade (Section 2.4); lymph node counts and lymphadenectomy describe the surgical procedure; and hormone receptor status is determined on the hysterectomy specimen and is not available preoperatively (Section 2.1). Receptor status is reported as assessed, not assessed and no record rather than as positive and negative, because the coding contains no negative category and every assessed tumor was receptor-positive; no significance test is therefore reported. Tests: Welch t (postoperative day-1 albumin, total lymph nodes harvested); Student’s t (pelvic lymph nodes harvested); Mann–Whitney U (length of stay, para-aortic lymph nodes harvested); Pearson’s chi-square (lymphadenectomy). * *p* < 0.05; *** *p* < 0.001.

**Table 4:** Discrimination and calibration of the prespecified elastic-net model, by feature set and cohort.

Cohort	Feature Set	n (Events)	AUC-ROC (95% CI)	AUC-PR	Balanced Accuracy	MCC	Brier	Prevalence-Only Brier	CITL	Slope
Full cohort	Set A	224 (59)	0.533 (0.443–0.621)	0.300	0.514	0.025	0.251	0.194	−1.02	0.03
	Set A-full	224 (59)	0.520 (0.432–0.609)	0.302	0.511	0.020	0.249	0.194	−1.00	0.08
	Set B	181 (48)	0.687 (0.590–0.772)	0.461	0.614	0.226	0.225	0.195	−0.97	1.58
	Set C	181 (48)	0.663 (0.562–0.752)	0.439	0.654	0.293	0.230	0.195	−0.99	1.36
Endometrioid only	Set A	182 (20)	0.592 (0.463–0.718)	0.142	0.559	0.074	0.221	0.098	−1.98	0.31
	Set A-full	182 (20)	0.556 (0.436–0.674)	0.127	0.531	0.039	0.224	0.098	−1.98	0.22
	Set B	147 (17)	0.390 (0.257–0.524)	0.095	0.454	−0.108	0.250	0.102	−2.01	−5.49
	Set C	147 (17)	0.463 (0.328–0.601)	0.109	0.479	−0.027	0.234	0.102	−1.93	−0.28
Receptor assessed	Set A	159 (28)	0.605 (0.481–0.723)	0.267	0.560	0.096	0.234	0.145	−1.49	0.36
	Set A-full	159 (28)	0.594 (0.473–0.712)	0.250	0.569	0.114	0.233	0.145	−1.47	0.51
	Set B	132 (24)	0.627 (0.485–0.765)	0.386	0.602	0.181	0.229	0.149	−1.45	1.82
	Set C	132 (24)	0.624 (0.474–0.762)	0.342	0.618	0.191	0.220	0.149	−1.39	0.86

Estimation procedure. Set A, reduced inflammatory panel (*p* = 8); Set A-full, full inflammatory panel (*p* = 14); Set B, preoperative clinical variables (*p* = 3); Set C, Sets A and B combined (*p* = 11); the variables of each set are listed in Section 2.4. Repeated nested cross-validation, twenty repetitions of a stratified five-fold outer loop with a stratified five-fold inner loop; 95% confidence intervals from 5000 stratified bootstrap resamples of pooled out-of-fold predictions. Classification thresholds were determined by the Youden index within each training fold and applied to the corresponding outer test fold. MCC, Matthews correlation coefficient; CITL, calibration-in-the-large; Slope, calibration slope. Calibration values are those of the raw predictions; after logistic recalibration, the intercept is 0 and the slope 1 by construction and the Brier score falls below the prevalence-only value in every row.

**Table 5:** Feature attributions. Mean absolute SHAP values are on the log-odds scale; standardized coefficients are those of the prespecified elastic-net model.

Feature Set (Model)	Feature	Mean |SHAP| (Log-Odds)	Elastic-Net Standardized Coefficient
Set B—clinical (mean |SHAP|: exploratory tree model; coefficients: prespecified elastic net)	Age	0.668	+0.612
	Preoperative albumin	0.338	−0.086
	CA-125	0.315	+0.248
Set A—inflammatory (exploratory tree model)	ELR	0.425	—
	NLR	0.256	—
	PIV	0.233	—
	LMR	0.226	—
	Monocytes	0.191	—
	Lymphocytes	0.185	—
	Platelets	0.183	—
	Neutrophils	0.158	—

Attribution procedure. SHAP values were computed with TreeExplainer using model_output = “raw” and are therefore on the log-odds scale; values are the mean absolute contribution across patients. Standardized coefficients are those of the prespecified elastic-net model fitted to standardized predictors. These attributions describe how each model uses its inputs and are not measures of discrimination; performance estimates throughout are those obtained by nested cross-validation (Table 4). Attributions for the inflammatory set are those of the exploratory tree model and are reported for descriptive purposes only; an em-dash indicates that no elastic-net coefficient applies, the inflammatory attributions being those of a tree model.

## Data Availability

The raw data supporting the conclusions of this article will be made available by the authors on request. The underlying patient-level data are pseudonymized rather than anonymized and cannot be shared in that form; a fully anonymized extract, from which the linkage key has been destroyed, can be made available upon reasonable request and subject to approval by the institutional ethics committee. The complete analysis code, including the corrected feature definitions, the three-cohort structure, and the scripts used to generate all tables and figures reported in the manuscript, is publicly available at: https://github.com/esra0709/endometrial-cancer-grade-ml (accessed on 10 September 2026).

## Supplementary Materials

The following supporting information can be downloaded at: https://www.mdpi.com/article/10.3390/diagnostics16182987/s1, Table S1: Methodological appraisal of the primary studies cited in the Introduction, against three criteria [6,7,8,9,10,11,12]; Table S2a: Missing values for each candidate predictor in the 225-patient analytic cohort; Table S2b: Joint missingness pattern for preoperative albumin, CA-125 and hormone receptor status; Table S2c: Included versus excluded patients (complete-case population of the preoperative clinical set); Table S3: All four feature sets evaluated in the common complete-case population of each cohort; Table S4: Retention frequency of each variable when the collinearity reduction is repeated independently within each of 25 outer training folds; Table S5a: Histologic subtype by tumor grade; Table S5b: Discrimination of nine classifiers across the four feature sets, full cohort; Table S6: Full specification of the prespecified primary model, generated from the analysis script; Table S7: Pattern of estrogen and progesterone receptor assessment by grade and histology; Table S8: One-sided non-superiority test of each inflammatory index and of both inflammatory models against a discrimination margin of 0.70; Table S9: Effect of the hyperparameter selection rule on the prespecified model; Table S10: Clinical and combined models refitted on all patients using multiple imputation by chained equations within the cross-validation folds.

## References

[1] Siegel R.L., Miller K.D., Jemal A. (2020). Cancer statistics, 2020. CA Cancer J. Clin..

[2] Creutzberg C.L., Nout R.A., Lybeert M.L., Wárlám-Rodenhuis C.C., Jobsen J.J., van de Poll-Franse L.V., van Putten W.L. (2011). Fifteen-year radiotherapy outcomes of the PORTEC-1 trial for endometrial carcinoma. Int. J. Radiat. Oncol. Biol. Phys..

[3] Amant F., Moerman P., Neven P., Timmerman D., Van Limbergen E., Vergote I. (2005). Endometrial cancer. Lancet.

[4] Göksedef B.P., Akbayır Ö., Corbacıoğlu A., Güraslan H., Sencan F., Erol O., Cetin A. (2012). Comparison of preoperative endometrial biopsy grade and final pathologic diagnosis in patients with endometrioid endometrial cancer. J. Turk. Ger. Gynecol. Assoc..

[5] Sia T.Y., Kraiem S., Rosalik K., Haney K., Schlappe B., Schiavone M.B., Dagher C., Ducie J., Eriksson A.G.Z., Makker V. (2026). Clinical behavior of FIGO stage I endometrioid endometrial adenocarcinoma diagnosed as high grade on pre-operative biopsy and low grade on hysterectomy specimen. Int. J. Gynecol. Cancer.

[6] Leng J., Wu F., Zhang L. (2022). Prognostic significance of pretreatment neutrophil-to-lymphocyte ratio, platelet-to-lymphocyte ratio, or monocyte-to-lymphocyte ratio in endometrial neoplasms: A systematic review and meta-analysis. Front. Oncol..

[7] Qin L. (2024). The predictive value of NLR, PLR and MLR in the differential diagnosis of benign uterine diseases and endometrial malignant tumors. Discov. Oncol..

[8] Ji P., He J. (2024). Prognostic value of pretreatment systemic immune-inflammation index in patients with endometrial cancer: A meta-analysis. Biomark. Med..

[9] Guo F., Peng H., Hu H., Liu J. (2025). Prognostic value of systemic immune-inflammation index in patients with gynecological tumors: A systematic review and meta-analysis. Am. J. Reprod. Immunol..

[10] Kirsch-Mangu A.T., Țîpcu A., Gâta V.A., Pop D.C., Fekete Z., Irimie A., Kubelac P.M. (2025). Neutrophil to lymphocyte ratio a prognostic tool in endometrial cancer among classical prognostic factors. Diagnostics.

[11] Pulur A., Gezer Ş., Duman Öztürk S., Eser M.D. (2025). Effects of neutrophil-to-lymphocyte ratio, tumor-infiltrating lymphocyte and neutrophil scores on prognosis in low-risk endometrial cancer. Eur. J. Gynaecol. Oncol..

[12] Ronsini C., Iavarone I., Vastarella M.G., Della Corte L., Andreoli G., Bifulco G., Cobellis L., de Franciscis P. (2025). Additional risk factors lead to a measurable inflammatory response in stage I endometrial cancer—A prospective multicentric observational study. Indian J. Surg. Oncol..

[13] Bhardwaj V., Sharma A., Parambath S.V., Gul I., Zhang X., Lobie P.E., Qin P., Pandey V. (2022). Machine learning for endometrial cancer prediction and prognostication. Front. Oncol..

[14] Lai H., Wu Q., Weng Z., Lyu G., Yang W., Ye F. (2026). Development of a high-performance ultrasound prediction model for the diagnosis of endometrial cancer: An interpretable XGBoost algorithm utilizing SHAP. J. Ultrasound Med..

[15] Yasar S., Yagin F.H., Melekoglu R., Ardigò L.P. (2024). Integrating proteomics and explainable artificial intelligence: A comprehensive analysis of protein biomarkers for endometrial cancer diagnosis and prognosis. Front. Mol. Biosci..

[16] WHO Classification of Tumours Editorial Board (2020). Female Genital Tumours.

[17] von Elm E., Altman D.G., Egger M., Pocock S.J., Gøtzsche P.C., Vandenbroucke J.P. (2007). The Strengthening the Reporting of Observational Studies in Epidemiology (STROBE) statement: Guidelines for reporting observational studies. Lancet.

[18] Collins G.S., Moons K.G.M., Dhiman P., Riley R.D., Beam A.L., Van Calster B., Ghassemi M., Liu X., Reitsma J.B., van Smeden M. (2024). TRIPOD+AI statement: Updated guidance for reporting clinical prediction models that use regression or machine learning methods. BMJ.

[19] Hu B., Yang X.-R., Xu Y., Sun Y.-F., Sun C., Guo W., Zhang X., Wang W.-M., Qiu S.-J., Zhou J. (2014). Systemic immune-inflammation index predicts prognosis of patients after curative resection for hepatocellular carcinoma. Clin. Cancer Res..

[20] Fucà G., Guarini V., Antoniotti C., Morano F., Moretto R., Corallo S., Marmorino F., Lonardi S., Rimassa L., Sartore-Bianchi A. (2020). The pan-immune-inflammation value is a new prognostic biomarker in metastatic colorectal cancer. Br. J. Cancer.

[21] Hastie T., Tibshirani R., Friedman J. (2009). The Elements of Statistical Learning.

[22] Benjamini Y., Hochberg Y. (1995). Controlling the false discovery rate: A practical and powerful approach to multiple testing. J. R. Stat. Soc. Ser. B.

[23] DeLong E.R., DeLong D.M., Clarke-Pearson D.L. (1988). Comparing the areas under two or more correlated receiver operating characteristic curves: A nonparametric approach. Biometrics.

[24] Hosmer D.W., Lemeshow S. (2000). Applied Logistic Regression.

[25] Berger R.L., Hsu J.C. (1996). Bioequivalence trials, intersection-union tests and equivalence confidence sets. Stat. Sci..

[26] van der Ploeg T., Austin P.C., Steyerberg E.W. (2014). Modern modelling techniques are data hungry: A simulation study for predicting dichotomous endpoints. BMC Med. Res. Methodol..

[27] Riley R.D., Collins G.S. (2023). Stability of clinical prediction models developed using statistical or machine learning methods. Biom. J..

[28] Van Calster B., McLernon D.J., van Smeden M., Wynants L., Steyerberg E.W. (2019). Calibration: The Achilles heel of predictive analytics. BMC Med..

[29] Vickers A.J., Elkin E.B. (2006). Decision curve analysis: A novel method for evaluating prediction models. Med. Decis. Mak..

[30] Hanley J.A., McNeil B.J. (1982). The meaning and use of the area under a receiver operating characteristic (ROC) curve. Radiology.

[31] Concin N., Matias-Guiu X., Cibula D., Colombo N., Creutzberg C.L., Ledermann J., Mirza M.R., Vergote I., Abu-Rustum N.R., Bosse T. (2025). ESGO–ESTRO–ESP guidelines for the management of patients with endometrial carcinoma: Update 2025. Lancet Oncol..

[32] Vickers A.J., van Calster B., Steyerberg E.W. (2019). A simple, step-by-step guide to interpreting decision curve analysis. Diagn. Progn. Res..

[33] Lundberg S.M., Lee S.-I. (2017). A unified approach to interpreting model predictions. Adv. Neural Inf. Process. Syst..

[34] Riley R.D., Snell K.I.E., Ensor J., Burke D.L., Harrell F.E., Moons K.G.M., Collins G.S. (2019). Minimum sample size for developing a multivariable prediction model: PART II—Binary and time-to-event outcomes. Stat. Med..

[35] Riley R.D., Ensor J., Snell K.I.E., E Harrell F., Martin G.P., Reitsma J.B., Moons K.G.M., Collins G., van Smeden M. (2020). Calculating the sample size required for developing a clinical prediction model. BMJ.

[36] Tsegaye B., Snell K.I.E., Archer L., Kirtley S., Riley R.D., Sperrin M., Van Calster B., Collins G.S., Dhiman P. (2025). Larger sample sizes are needed when developing a clinical prediction model using machine learning in oncology: Methodological systematic review. J. Clin. Epidemiol..

[37] van Royen F.S., Weerts H.J.P., de Hond A.A.H., Geersing G.J., Rutten F.H., Moons K.G.M., van Smeden M. (2026). In humble defense of unexplainable black box prediction models in healthcare. J. Clin. Epidemiol..

[38] Vickers A.J., Holland F. (2021). Decision curve analysis to evaluate the clinical benefit of prediction models. Spine J..

