# Supplementary material for: Hematological Versus Clinical Predictors of Tumor Grade in Endometrial Cancer: A Comparative Machine Learning Study with SHAP-Based Explainability
Source: Diagnostics (Basel). 2026 Sep 15;16(18):2987. doi: 10.3390/diagnostics16182987 (PMC13605369; doi:10.3390/diagnostics16182987)
Supplement: Supplementary file 1 [file diagnostics-16-02987-s001.zip › diagnostics-4511298-supplementary.pdf]

Supplementary Material

Hematological Versus Clinical Predictors of Tumor Grade in Endometrial Cancer: A Comparative Machine Learning Study with SHAP-Based Explainability

Esra Akaydın Gültürk, Şerife Özlem Genç · Manuscript diagnostics-4511298

All results are those of the revised analysis: estrogen and progesterone receptor status excluded from every feature set, a prespecified elastic-net logistic regression as the primary model, and every analysis repeated in three cohorts.

Appraisal of the cited primary studies

Table S1. Methodological appraisal of the primary studies cited in the Introduction, against three criteria.

Study (reference)	Question addressed	n	Indices tested	Correction for multiple comparisons	Multicollinearity assessed	Nested or external validation
Qin [7]	Malignant versus benign	154 (80 / 74)	NLR, PLR, MLR	No	Not reported	No
Kirsch-Mangu et al. [10]	Prognosis	398	NLR	Not applicable	Not reported	No
Pulur et al. [11]	Prognosis	233	NLR (with TIL and TIN scores)	No	Not reported	No
Ronsini et al. [12]	Clinicopathological association	163	NLR, PLR, MLR	No	Not reported	No

The three meta-analyses cited in the same paragraph [6,8,9] pool published effect estimates rather than analyzing patient-level data, and do not develop predictive models; none of the three criteria applies to them, and they are therefore not appraised here. Question addressed is one of prognosis, malignant versus benign, clinicopathological association, or preoperative discrimination of G3 from G1–G2; no cited study addresses the last of these. "No" denotes that the methods were read and describe a different procedure; "not reported" that the full text was read and the criterion is not mentioned; "not applicable" that only one index was tested, so multiplicity across indices does not arise. Correction for multiple comparisons refers to adjustment across the indices tested. Multicollinearity refers to formal assessment (variance inflation, tolerance, correlation structure or condition number) among indices sharing neutrophil, lymphocyte or platelet counts. A single train–test split or an in-sample receiver operating characteristic analysis is recorded as "no".

Missing data

Table S2a. Missing values for each candidate predictor in the 225-patient analytic cohort.

Variable	Missing (n)	Missing (%)
Age	0	0.0
CA-125	5	2.2
Preoperative albumin	44	19.6
ER assessed (0 code = not assessed)	29	12.9
PR assessed	29	12.9
eos	1	0.4
lym	1	0.4
mon	1	0.4
neu	1	0.4
WBC	1	0.4
plt	1	0.4

Receptor status is reported for completeness only; it was not used as a predictor in any model (Section 2.1).

Table S2b. Joint missingness pattern for preoperative albumin, CA-125 and hormone receptor status.

Preoperative albumin missing	CA-125 missing	Receptor status missing	Patients (n)
no	no	no	157
no	no	yes	24

Preoperative albumin missing	CA-125 missing	Receptor status missing	Patients (n)
yes	no	no	36
yes	no	yes	3
yes	yes	no	3
yes	yes	yes	2

The pattern is not monotone, so a single missingness mechanism cannot be assumed.

Table S2c. Included versus excluded patients (complete-case population of the preoperative clinical set).

Variable	Included (median)	Excluded (median)	n included	n excluded	p (Mann–Whitney U)
Age	60.000	58.000	181	44	0.725
CA-125	19.300	12.800	181	39	0.002
NLR	2.931	2.343	181	44	0.043
ELR	0.051	0.053	181	44	0.444
PIV	365.940	256.413	181	44	0.125
Lymphocytes	1.720	2.130	181	43	0.0
Platelets	272.000	295.000	181	43	0.084
G3 proportion	0.265	0.250	181	44	0.988
Endometrioid proportion	0.812	0.818	181	44	1.0

Selection is associated with CA-125, lymphocyte count and NLR, but not with the outcome (G3 proportion, p = 0.988) or with histology (endometrioid proportion, p = 1.000). For the last two rows the proportion is given rather than a median.

Common complete-case population

Table S3. All four feature sets evaluated in the common complete-case population of each cohort.

Cohort	Feature set	n	Events	AUC	95% CI	AUC-PR	Balanced accuracy	MCC	Brier	Prevalence-only Brier
Full cohort	Set A	181	48	0.549	0.452–0.648	0.306	0.556	0.098	0.252	0.195
Full cohort	Set A-full	181	48	0.557	0.461–0.654	0.317	0.541	0.073	0.249	0.195
Full cohort	Set B	181	48	0.687	0.590–0.771	0.461	0.614	0.226	0.225	0.195
Full cohort	Set C	181	48	0.663	0.562–0.752	0.439	0.654	0.293	0.230	0.195
Endometrioid only	Set A	147	17	0.573	0.445–0.695	0.135	0.585	0.109	0.225	0.102
Endometrioid only	Set A-full	147	17	0.530	0.396–0.658	0.124	0.481	−0.024	0.227	0.102
Endometrioid only	Set B	147	17	0.390	0.257–0.523	0.095	0.454	−0.108	0.250	0.102
Endometrioid only	Set C	147	17	0.463	0.328–0.601	0.109	0.479	−0.027	0.234	0.102
ER assessed	Set A	132	24	0.624	0.503–0.742	0.240	0.609	0.169	0.233	0.149
ER assessed	Set A-full	132	24	0.626	0.502–0.743	0.270	0.565	0.102	0.228	0.149
ER assessed	Set B	132	24	0.627	0.484–0.765	0.386	0.602	0.181	0.229	0.149
ER assessed	Set C	132	24	0.624	0.474–0.762	0.342	0.618	0.191	0.220	0.149

Collinearity reduction repeated within folds

Table S4. Retention frequency of each variable when the collinearity reduction is repeated independently within each of 25 outer training folds.

Variable	Retained in folds (%)	In the published Set A
lym	100%	yes
mon	100%	yes
neu	100%	yes
plt	100%	yes
NLR	100%	yes

Variable	Retained in folds (%)	In the published Set A
ELR	100%	yes
LMR	100%	yes
PIV	64%	yes

The fold-wise reduction reproduces the published Set A. Maximum variance inflation factor in Set A: 8.36.

Histologic subtype

Table S5a. Histologic subtype by tumor grade.

Subtype	G1	G2	G3	Total	G3 (%)
Clear cell	1	0	5	6	83.3
Endometrioid	130	33	20	183	10.9
Mixed	0	0	10	10	100.0
Mucinous	1	0	0	1	0.0
Serous	0	0	21	21	100.0
Squamous	1	0	1	2	50.0
Undifferentiated	0	0	2	2	100.0
All	133	33	59	225	26.2

Of 59 high-grade events, 39 (66.1%) occurred in non-endometrioid tumors, and 92.9% of non-endometrioid tumors were G3. Serous, clear-cell and undifferentiated carcinomas are high-grade by definition.

Exploratory comparison among nine classifiers

Table S5b. Discrimination of nine classifiers across the four feature sets, full cohort.

Algorithm	Feature set	p	n	Events	AUC	95% CI
LogReg_EN	Set A	8	224	59	0.531	0.445–0.619
LogReg_EN	Set A-full	14	224	59	0.521	0.432–0.610
LogReg_EN	Set B	3	181	48	0.684	0.588–0.778
LogReg_EN	Set C	11	181	48	0.662	0.567–0.754
SVM_RBF	Set A	8	224	59	0.442	0.356–0.527
SVM_RBF	Set A-full	14	224	59	0.436	0.352–0.521
SVM_RBF	Set B	3	181	48	0.648	0.553–0.741
SVM_RBF	Set C	11	181	48	0.643	0.546–0.736
KNN	Set A	8	224	59	0.469	0.379–0.560
KNN	Set A-full	14	224	59	0.509	0.423–0.594
KNN	Set B	3	181	48	0.613	0.508–0.717
KNN	Set C	11	181	48	0.646	0.560–0.731
RandomForest	Set A	8	224	59	0.541	0.450–0.629
RandomForest	Set A-full	14	224	59	0.560	0.466–0.649
RandomForest	Set B	3	181	48	0.617	0.514–0.713
RandomForest	Set C	11	181	48	0.641	0.546–0.737
ExtraTrees	Set A	8	224	59	0.506	0.412–0.600
ExtraTrees	Set A-full	14	224	59	0.519	0.427–0.612
ExtraTrees	Set B	3	181	48	0.656	0.554–0.752
ExtraTrees	Set C	11	181	48	0.643	0.548–0.736
GradBoost	Set A	8	224	59	0.587	0.497–0.671
GradBoost	Set A-full	14	224	59	0.593	0.502–0.682
GradBoost	Set B	3	181	48	0.578	0.478–0.672
GradBoost	Set C	11	181	48	0.602	0.503–0.695
XGBoost	Set A	8	224	59	0.544	0.456–0.626

Algorithm	Feature set	p	n	Events	AUC	95% CI
XGBoost	Set A-full	14	224	59	0.578	0.488–0.665
XGBoost	Set B	3	181	48	0.595	0.495–0.692
XGBoost	Set C	11	181	48	0.627	0.528–0.721
LightGBM	Set A	8	224	59	0.540	0.448–0.629
LightGBM	Set A-full	14	224	59	0.560	0.472–0.649
LightGBM	Set B	3	181	48	0.544	0.442–0.639
LightGBM	Set C	11	181	48	0.600	0.502–0.697
CatBoost	Set A	8	224	59	0.569	0.477–0.659
CatBoost	Set A-full	14	224	59	0.571	0.476–0.661
CatBoost	Set B	3	181	48	0.581	0.482–0.675
CatBoost	Set C	11	181	48	0.622	0.526–0.718

Repeated nested cross-validation, five repetitions of a stratified five-fold outer loop with a stratified three-fold inner loop; 95% confidence intervals from 2000 stratified bootstrap resamples. The algorithm with the highest value within a feature set is identified on out-of-fold performance and therefore carries selection optimism; the maximum across algorithms is an optimistically biased estimate of the performance of any one of them. In the preoperative clinical set the best of the nine is the elastic net itself (0.684), against 0.687 for the prespecified primary analysis.

Hyperparameter specification

Table S6. Full specification of the prespecified primary model, generated from the analysis script.

Item	Value
Model	Elastic-net logistic regression (prespecified primary)
Penalty grid (C)	np.logspace(−5, 1, 20)
Mixing parameter grid (l1_ratio)	[0.0, 0.5, 1.0]
Configurations searched	60
Search strategy	exhaustive grid (GridSearchCV)
Refit rule	best
Inner resampling	stratified 5-fold
Outer resampling	stratified 5-fold
Outer repetitions	20
Tuning criterion	roc_auc
Class imbalance	class_weight='balanced'
Imputation	fold-wise median (no-op in complete-case sets)
Scaling	StandardScaler, fitted within fold
Classification threshold	Youden J, selected WITHIN each training fold
Solver	saga, max_iter=20000
Random seed	42
Software versions	sklearn 1.6.1, scipy 1.16.3, numpy 2.1.3, statsmodels 0.14.6, shap 0.52.0

Hormone receptor assessment pattern

Table S7. Pattern of estrogen and progesterone receptor assessment by grade and histology.

Marker	Category	G1–G2 (n)	G3 (n)	G3 (%)	Endometrioid (%)
ER	assessed, positive	131	28	17.6	87.4
ER	code 0 = NOT ASSESSED	14	23	62.2	51.4
ER	no record	21	8	27.6	86.2
PR	assessed, positive	128	27	17.4	88.4
PR	code 0 = NOT ASSESSED	17	24	58.5	51.2
PR	no record	21	8	27.6	86.2

The source variables are ten-level ordinal categories of staining percentage (1 = 10–20% through 9 = 90–100%, 10 = "only positive"). There is no negative category, and every patient in whom receptor status was assessed was receptor-positive; the code 0 denotes "not assessed". No significance test is reported, since such a test would compare grade with whether the assay was requested rather than with receptor status.

Non-superiority testing

Table S8. One-sided non-superiority test of each inflammatory index and of both inflammatory models against a discrimination margin of 0.70.

Index or model	n	AUC	95% CI	One-sided upper bound	Null rejected
NLR	225	0.505	0.418–0.590	0.577	yes
dNLR	224	0.520	0.439–0.604	0.591	yes
PLR	225	0.533	0.446–0.624	0.609	yes
MLR	225	0.537	0.454–0.625	0.611	yes
LMR	224	0.535	0.449–0.619	0.607	yes
SII	225	0.500	0.411–0.591	0.577	yes
SIRI	225	0.502	0.418–0.590	0.575	yes
PIV	225	0.514	0.427–0.603	0.590	yes
ELR	225	0.626	0.543–0.705	0.694	yes
Set A	224	0.532	0.443–0.621	0.607	yes
Set A-full	224	0.520	0.432–0.609	0.595	yes

Null hypothesis: the area under the curve is at least 0.70. The margin was adopted post hoc and this analysis is reported as exploratory. Rejection of the family null requires every component test to reject (intersection–union test). The bound for ELR is 0.694, six thousandths below the margin. Confidence intervals and the one-sided upper bound are percentile bootstrap values (5000 stratified resamples), consistent with the bound itself; the intervals reported in Table 3 of the main text are DeLong intervals, which differ in the third decimal.

Hyperparameter refit rule

Table S9. Effect of the hyperparameter selection rule on the prespecified model.

Feature set	Refit rule	AUC	Brier	Median C selected	Folds with C at grid minimum (%)
Set A	best inner-loop score	0.524	0.253	0.546	4%
Set A	one standard error	0.497	0.255	0.000	64%
Set A-full	best inner-loop score	0.507	0.251	0.062	12%
Set A-full	one standard error	0.484	0.254	0.000	64%
Set B	best inner-loop score	0.690	0.224	0.127	4%
Set B	one standard error	0.642	0.248	0.000	88%
Set C	best inner-loop score	0.670	0.231	0.030	0%
Set C	one standard error	0.653	0.244	0.000	52%

Under the one-standard-error rule the penalty parameter is selected at the lower bound of the search grid in 52% to 88% of folds, shrinking the coefficients almost to zero. The primary analysis therefore refits on the best inner-loop score. Values in this table are from the internal comparison and differ in the third decimal from Table 3, which averages over twenty repetitions.

Multiple imputation

Table S10. Clinical and combined models refitted on all patients using multiple imputation by chained equations within the cross-validation folds.

Cohort	Feature set	n	Events	AUC	95% CI	Brier
Full cohort	Set B	225	59	0.693	0.608–0.774	0.222
Full cohort	Set C	225	59	0.691	0.606–0.773	0.225
Endometrioid only	Set B	183	20	0.397	0.255–0.546	0.250

Cohort	Feature set	n	Events	AUC	95% CI	Brier
Endometrioid only	Set C	183	20	0.517	0.388–0.647	0.228
ER assessed	Set B	159	28	0.639	0.498–0.771	0.228
ER assessed	Set C	159	28	0.656	0.523–0.782	0.224

The imputation model was fitted inside each training fold, so held-out patients contributed nothing to imputation. Compare with the complete-case values in Table 3: full cohort Set B 0.687, endometrioid Set B 0.390, receptor-assessed Set B 0.627.
